# P-1845. Hepatitis C Care Continuum among People Who Inject Drugs at a Safety-Net Hospital in a Large U.S. City

**DOI:** 10.1093/ofid/ofaf695.2014

**Published:** 2026-01-11

**Authors:** Elizabeth Golding, Sophia Goldin, Kevin Kamis, Theodore Yoder, Sarah E Rowan

**Affiliations:** University of Colorado School of Medicine, Aurora, Colorado; University of Colorado School of Medicine, Aurora, Colorado; Denver Health and Hospital Authority, Denver, Colorado; University of Colorado School of Medicine, Aurora, Colorado; Denver Public Health, Denver, CO

## Abstract

**Background:**

Direct acting antivirals (DAAs) are over 95% effective at curing hepatitis C virus (HCV) infection, yet treatment rates remain low among people who inject drugs (PWID). This study characterizes HCV treatment among PWID at a safety net hospital in a large city. We conducted a retrospective chart review to quantify the number of PWID who progress through each stage of the care continuum and identify major gaps and disparities in care.

**Figure:**
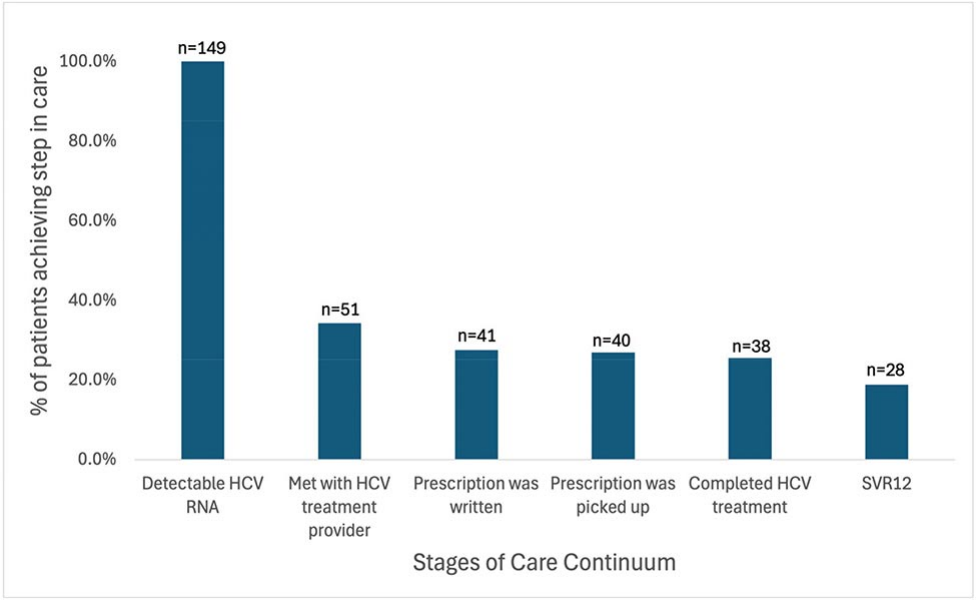
Number of Patients Completing Each Stage of the Hepatitis C Care Continuum

**Figure:**
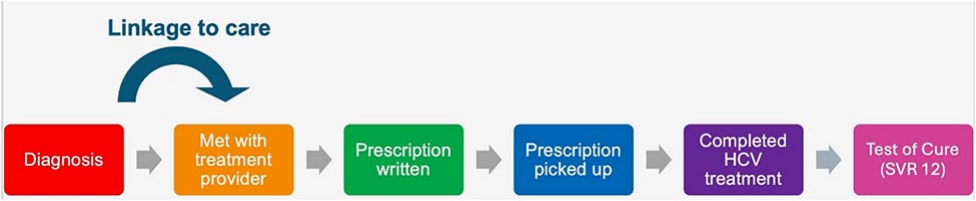
Hepatitis C Care Continuum

**Methods:**

A retrospective chart review was performed of adult patients seen at Denver Health with detectable HCV RNA results in the medical record between November 1, 2021 and October 31, 2022. Individuals with evidence of injection drug use (IDU) between May 1, 2021 (6 months prior to index HCV RNA test result) and October 31, 2022 were included in the analysis. The furthest step of the HCV treatment care continuum completed for each patient was identified from the medical records extending through October 2023. Stages included 1) detectable HCV RNA, 2) met HCV treatment provider, 3) prescription was written, 4) prescription was dispensed, 5) completed HCV treatment, and 6) evidence of sustained virologic response 12 weeks after the end of treatment (SVR12). Patient characteristics were compared among the groups of patients who did and did not link to care, and reasons for loss to follow-up were reviewed.

**Figure:**
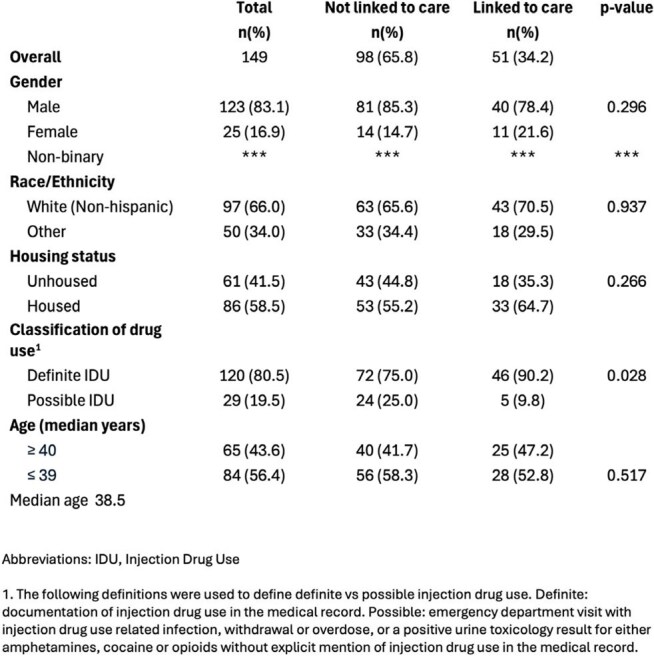
Patient Characteristics

**Results:**

Of the 149 PWID diagnosed with HCV, 34.2% (n=51) met with an HCV treatment provider, 25.5% (n=38) completed treatment and 18.8% (n=28) had documented SVR12. Of the 51 who met with an HCV provider, 74.5% completed treatment and 55% had documented SVR12. Gender, race/ethnicity, and housing status were not significantly correlated with linkage to care. While 74.5% of all study participants did not complete HCV care, only 12.8% had documented reasons in the medical record. Reasons for not completing care included incarceration, terminal illness, death, moving out of state, provider or patient decision to postpone care, and treatment at an outside facility.

**Conclusion:**

This study’s findings indicate that the most difficult step in achieving cure of HCV among PWID is accessing an HCV treatment provider after diagnosis, with a majority completing treatment once they met with a treatment provider.

**Disclosures:**

All Authors: No reported disclosures

